# The novel antimicrobial peptide PXL150 in the local treatment of skin and soft tissue infections

**DOI:** 10.1007/s00253-012-4439-8

**Published:** 2012-10-04

**Authors:** Emma Myhrman, Joakim Håkansson, Kerstin Lindgren, Camilla Björn, Veronika Sjöstrand, Margit Mahlapuu

**Affiliations:** Pergamum AB, Arvid Wallgrens Backe 20, 413 46 Gothenburg, Sweden

**Keywords:** Antimicrobial peptide, Wound infection, Antibiotic resistance, *Staphylococcus aureus*, MRSA

## Abstract

Dramatic increase in bacterial resistance towards conventional antibiotics emphasises the importance to identify novel, more potent antimicrobial therapies. Antimicrobial peptides (AMPs) have emerged as a promising new group to be evaluated in therapeutic intervention of infectious diseases. Here we describe a novel AMP, PXL150, which demonstrates in vitro a broad spectrum microbicidal action against both Gram-positive and Gram-negative bacteria, including resistant strains. The potent microbicidal activity and broad antibacterial spectrum of PXL150 were not associated with any hemolytic activity. *Staphylococcus aureus* and methicillin-resistant *S. aureus* (MRSA) failed to develop resistance towards PXL150 during continued selection pressure. PXL150 caused a rapid depolarisation of cytoplasmic membrane of *S. aureus*, and dissipating membrane potential is likely one mechanism for PXL150 to kill its target bacteria. Studies in human cell lines indicated that PXL150 has anti-inflammatory properties, which might be of additional benefit. PXL150 demonstrated pronounced anti-infectious effect in an in vivo model of full thickness wounds infected with MRSA in rats and in an ex vivo model of pig skin infected with *S. aureus*. Subcutaneous or topical application of the peptide in rats did not lead to any adverse reactions. In conclusion, PXL150 may constitute a new therapeutic alternative for local treatment of infections, and further studies are warranted to evaluate the applicability of this AMP in clinical settings.

**Electronic supplementary material:**

The online version of this article (doi:10.1007/s00253-012-4439-8) contains supplementary material, which is available to authorized users.

## Introduction

During the past decades, there has been a dramatic increase in the number of patients presenting skin and soft tissue infections (SSTIs) manifesting as furuncles, carbuncles, abscesses, impetigo or cellulitis, in ambulatory settings (Hersh et al. 2008; Pallin et al. 2008). The predominant causative bacterium in this type of infections is *Staphylococcus aureus*. *S. aureus* is a Gram-positive bacterial species commonly found in healthy people, yet it is known to cause a variety of diseases ranging from mild superficial skin infections to life-threatening disorders (Fey et al. 2003). Antibiotic resistance has long been a problem associated with *S. aureus.* The main challenge in the treatment of SSTIs is the rapid increase of patients with community-acquired strains of methicillin-resistant *S. aureus* (CA-MRSA). These patients do not exhibit any of the commonly accepted risk factors for hospital-associated MRSA. Furthermore, CA-MRSA isolates represent a different genetic entity than the hospital-associated variety (Naimi et al. 2003). The global epidemiology of CA-MRSA is heterogeneous regarding both overall frequency and clonal diversity. As part of the SENTRY Antimicrobial Surveillance Program, Moet and co-authors studied the epidemiology of all organisms associated with SSTIs in hospitalised patients over a 7-year period (1998–2004). A considerable variation in the MRSA rate was noted between countries and continents, with the overall rate highest in North America (35.9 %) compared with Latin America (29.4 %) and Europe (22.8 %) (Moet et al. 2007). In some geographic regions, a single clone of CA-MRSA dominates, e.g. the USA300 strain in the USA, whereas in other regions, multiple clones have been identified, e.g. there are more than 100 clones described in Australia (Chua et al. 2011). While *S. aureus* remains the dominant pathogen in SSTIs, Gram-negative bacteria are also frequently isolated. According to the SENTRY Antimicrobial Surveillance Program, *Pseudomonas aeruginosa* constituted greater than 10 % of the pathogens in SSTIs for all regions, ranking second in North America and Europe and third in Latin America, where *Escherichia coli* was ranked second (Moet et al. 2007). Notably, multidrug-resistant *P. aeruginosa* (resistant to four drug classes) in SSTIs had a calculated occurrence rate of 24.7 % in Latin America, 10.8 % in Europe and 3.2 % in North America, which indicates alarming increase in drug resistance also among Gram-negative bacteria (Moet et al. 2007).

With the steep increase in resistance development to conventional antibiotics, it is imperative to find more potent antimicrobial strategies, which are effective against resistant strains and have broad spectrum of activity. Antimicrobial peptides (AMPs), ancient molecules in the innate immune response of eukaryotes, have recently emerged as a promising new group to be evaluated in therapeutic intervention of infectious diseases (reviewed in Zasloff 2002). AMPs have activities against a broad range of bacteria and fungi. Interestingly, the development of resistance toward AMPs has occurred to a much lesser degree compared to conventional antibiotics as the mechanism of killing bacteria by AMPs usually involves attacking multiple hydrophobic and/or polyanionic targets (reviewed in Fjell et al. 2012). It has been shown that the antibacterial target of many AMPs is the cytoplasmic membrane of bacterial cells (Nakajima et al. 2003; Powers and Hancock 2003; Silvestro et al. 2000).

In this study, we present a new synthetic, water-soluble low molecular weight AMP, PXL150, with broad microbicidal activity against both Gram-positive and Gram-negative bacteria, including MRSA. Potential anti-infectious effect of PXL150 was studied in an in vivo excision wound model in rats and an ex vivo pig skin model. The data presented indicate an advantageous safety profile and efficacy pattern of PXL150, which warrants further clinical investigations of the potential of this peptide in the treatment of SSTIs.

## Materials and methods

### Peptides and antibiotics

PXL150 was purchased from Biopeptide Co., Inc. (San Diego, CA, USA) and Bachem AG (Bubendorf, Switzerland). The peptide was synthesised using Fmoc solid phase technology, the identity was checked by electrospray ionisation mass spectrometry and the purity was determined by HPLC. Mupirocin (Bactroban, 2 % ointment, GlaxoSmithKline, Brentford, UK) and fusidic acid (Fucidin, 2 % cream, LEO Pharma, Malmö, Sweden) were used as control antibiotics. In the in vitro resistance assay, gentamicin (G1914, Sigma-Aldrich, St. Louis, MO, USA) in powder form was used as antibiotic control.

### In vitro minimal microbicidal concentration assay

The microbicidal effect of PXL150 was tested against *S. aureus* (American Type Culture Collection (ATCC) 12600), MRSA (ATCC 33591), *Streptococcus pyogenes* (ATCC 12344), *Propionibacterium acnes* (ATCC 6919), *Staphylococcus epidermidis* (ATCC 12228), *E. coli* (ATCC 11775), *P. aeruginosa* (ATCC 15442), *Klebsiella pneumoniae* (Culture Collection, University of Gothenburg (CCUG) 59413, clinical isolate resistant to penicillins, cephalosporins, aztreonam and carbapenems, with the reference strain ATCC 13883), *Acinetobacter baumannii* (CCUG 58437, clinical isolate resistant to tobramycin, trimsulfa, ciprofloxacin, cefotaxim, ceftazidim, meropenem, pipera/tazobactam, with the reference strain ATCC 19606) and the yeast strains *Candida albicans* (ATCC 64549), *Candida parapsilosis* (ATCC 22019), *Candida glabrata* (CCUG 35267) and *Candida krusei* (ATCC 6258) using a minimal microbicidal concentration (MMC) assay. The experiments were performed using 0.037 % brain heart infusion (BHI) broth (Difco, BD Diagnostics, Franklin Lakes, NJ, USA). To mimic the physiological conditions in wounds, the microbicidal effect of PXL150 against *S. aureus* and MRSA was also tested in 50 % heat-inactivated (h.i.) simulated wound fluid (SWF) composed of 0.1 % peptone (Oxoid, Basingstoke, UK) and 10 % foetal bovine serum (FBS; PAA Laboratories GmbH, Pasching, Austria) in a 50:50 mixture.

Bacteria were cultured overnight in 3.7 % BHI broth on a shaker at 37 °C, 250 rpm. The culture was diluted 1:10 in fresh 3.7 % BHI and incubated for an additional 2 h to reach exponential phase growth. The bacterial suspension was centrifuged at 900 × *g* and the pellet was resuspended in 0.037 % BHI to a concentration of 10^7^ colony forming units (CFU)/ml, estimated by measuring optical density at 600 nm (OD_600_). The peptide was serially diluted by twofold steps to a final concentration range of 1.56 to 200 μg/ml in 0.037 % BHI or 50 % h.i. SWF. One hundred microliters of the peptide solutions was mixed with 5 μl bacterial suspension in the wells of a 96-well plate (Nunc 267334, Roskilde, Denmark) and incubated at 37 °C for 2 h. Five microliters of each suspension was aspirated, added as drops onto agar plates (Columbia agar, Oxoid, Basingstoke, UK) supplemented with 5 % defibrinated horse blood (Swedish National Veterinary Institute, Uppsala, Sweden) and incubated overnight at 37 °C. All samples were processed as duplicates. The minimal peptide concentration causing ≥99 % reduction of microorganisms was defined as the MMC_99_. The concentration of the bacterial suspension used in the assay was confirmed by viable count estimation on blood agar plates.

### Hemolytic assay

The hemolytic activity of PXL150 was determined using fresh human erythrocytes. The erythrocytes were separated from blood supplemented with EDTA by centrifugation at 1,000 × *g* for 5 min, washed three times with PBS (pH 7.3) by centrifugation at 1,000 × *g* for 5 min and resuspended in PBS to a final red blood cell (RBC) concentration of 10 % (*v*/*v*). The peptide was serially diluted in PBS to the final peptide concentration range of 1.56 to 200 μg/ml. Ten microliters of RBC suspension, 50 μl of peptide solution and 40 μl of PBS were transferred to the wells of a round bottom 96-well plate (Nunc 267334) and incubated for 1 h at 37 °C. The plate was centrifuged at 1,000 × *g* for 5 min and the supernatants were transferred to a flat bottom 96-well plate (Nunc 439454). The release of hemoglobin was monitored by measuring the absorbance of the supernatant at 540 nm. The absorbance obtained after treating erythrocytes with PBS (negative control) or 0.1 % Triton X-100 (positive control) were set as 0 and 100 % hemolysis, respectively. The percentage of hemolysis was calculated by using the following equation: % hemolysis = 100 % × (Abs − Abs_0 %_) / (Abs_100 %_ − Abs_0 %_).

### Multistep resistance assay

Bacterial suspensions of *S. aureus* (ATCC 29213) and MRSA (ATCC 33591) were prepared according to the protocol described above, and the bacterial concentration was adjusted to 10^8^ CFU/ml in 0.037 % BHI. The multistep resistance assay was performed as previously described (Kosowska-Shick et al. 2006). Shortly, peptide dilutions were prepared in a volume of 1 ml in twofold dilution steps, in a concentration range from 0.75 to 96 μg/ml in 0.37 % BHI. The initial concentration range was based on the minimal inhibitory concentration (MIC) value with six dilutions prepared below and two dilutions prepared above the MIC value. The same peptide concentration range was used during all passages and new peptide dilutions were prepared each day from stock solutions. Gentamicin in powder form (G1914, Sigma-Aldrich) was selected as a positive antibiotic control in these experiments since gentamicin has previously demonstrated potential for resistance development after serial passages of *S. aureus* (Houang and Greenwood 1977; Yamakawa et al. 2002). The positive antibiotic control was handled in the same way as the peptide except that the concentration range of gentamicin was 0.047 to 24 μg/ml. A volume of 10 μl (10^8^ CFU/ml) of the bacterial suspension was added to the peptide solutions, to the negative control (0.37 % BHI incubated without peptide) and to the positive antibiotic control. The suspensions were incubated at 37 °C for 24 h, and turbidity of the overnight cultures was determined by OD_600_ measurements. The corrected OD_600_ values of the overnight cultures were calculated by subtracting the OD_600_ of the peptide mixed with medium only, without any incubation step, from the OD_600_ measured on the overnight cultures. To create an optimal selection pressure, the overnight culture having the highest peptide concentration without marked reduction of bacterial viability (defined as OD_600_ value of >85 % compared to the value measured for the negative control sample) was further passed. The selected overnight culture was diluted in 0.37 % BHI broth to a concentration of 10^8^ CFU/ml. Ten microliters of the selected inoculum was transferred to vials with 1 ml peptide solution in 0.37 % BHI broth, and the suspensions were incubated at 37 °C for 24 h. The same procedure was performed with the positive antibiotic control. The remaining bacterial suspensions were centrifuged at 900 × *g* for 10 min, resuspended in a 50:50 mixture of 3.7 % BHI and 80 % glycerol and immediately frozen at −80 °C. The 24-h incubation and inoculation procedure described above was repeated for up to 21 days. To evaluate potential for resistance induction, the MMC_99_ assay was performed on the overnight cultures, as described above, at passages from day 0 to day 21.

### Cytoplasmic membrane depolarisation assay

The depolarisation of the cytoplasmic membrane of *S. aureus* (ATCC 29213) by PXL150 was assessed by measuring the disruption of the electrical potential gradients across the cytoplasmic membrane in intact bacteria using the membrane potential-sensitive cyanine dye DiSC_3_(5). DiSC_3_(5) accumulates in bacterial cells and self-quenches its own fluorescence. Thus, increase in fluorescence reading reflects release of the dye from bacterial cells caused by increase in membrane permeability. The assay was performed as previously described (Friedrich et al. 2000). Shortly, exponential phase bacteria were washed and resuspended in 1 ml of 10 mM Tris buffer pH 7.0 to a concentration of 4 × 10^7^ CFU/ml. One microliter of 0.4 mM DiSC_3_(5) (Sigma-Aldrich) was added to 1 ml bacterial suspension. Two minutes after the addition of the dye, the intensity had stabilised, indicating maximal uptake of the dye into bacterial cells, and 30 μl 3.3 M KCl was added to equilibrate the cytoplasmic and external potassium ion concentrations. One minute later, the peptide solution was added to yield a final concentration of PXL150 ranging from 0.625 to 160 μg/ml. The fluorescence intensity was monitored continuously in real time using a Cary Eclips Fluorescence Spectrophotometer (Varian, Palo Alto, CA, USA) for up to 15 min starting with the addition of the peptide. The fluorescence was monitored at an excitation wavelength of 650 nm and an emission wavelength of 670 nm. The most optimal wavelengths for monitoring fluorescence from DiSC_3_(5) (excitation at 650 nm and emission at 670 nm) were identified by an excitation spectrum scan between 550 and 660 nm and an emission spectrum scan between 630 and 750 nm. Ten-microliter aliquots were removed at time intervals indicated in order to measure viable counts. After sample collection, the aliquots were immediately diluted 10^3^-, 10^4^-, 10^5^- and 10^6^-fold 0.037 % BHI. Fifty microliters of the dilutions was spread onto blood agar plates and incubated overnight at 37 °C. The plates containing between 20 and 300 CFU were counted.

Prior to the experiments described above, it was observed that KCl or PXL150 did not exhibit any autofluorescence at the emission wavelength of 670 nm (Online Resource 1a). Moreover, addition of KCl did not show any inhibitory effect on the fluorescence signal of the dye, while the addition of the maximal concentration of PXL150 of 160 μg/ml led to a minor reduction in signal of approximately 10 % (Online Resource 1b). Thus, the assay was well suited to assess changes in fluorescence signal reflecting the membrane depolarising properties of PXL150.

### Assessment of anti-inflammatory effect in THP-1 cells

The human monocytic cell line THP-1 (ATCC TIB-202) was cultured in RPMI 1640 medium (PAA Laboratories GmbH) supplemented with 10 % FBS (PAA Laboratories GmbH), 1 mM sodium pyruvate (Sigma-Aldrich) and 20 mM 4-(2-hydroxyethyl)-1-piperazineethanesulfonic acid (HEPES, PAA Laboratories GmbH). The cell density was adjusted to 10^6^ cells/ml, and 100 μl of the cell suspension was added to the wells of a 96-well cell culture plate (Sarstedt, Nümbrecht, Germany). The cells were incubated with 10 ng/ml phorbol 12-myristate 13-acetate (PMA, Sigma-Aldrich) to differentiate the monocytes into macrophage-like cells. After 2 days, the cells were stimulated with 0.1 ng/ml lipopolysaccharide (LPS, *E. coli* serotype O55:B5, Sigma-Aldrich) in the medium specified above except that 10 % FBS was replaced with 5 % h.i. FBS. Titration experiments were previously performed to select the LPS concentration mediating a close to maximum release of cytokines after 6 h. Thirty minutes after the addition of LPS, PXL150 in a concentration range from 0.625 to 80 μM was added. The supernatants were collected after 6 h of incubation and frozen at −20 ***°***C. The samples were kept frozen until analysed for tumor necrosis factor alpha (TNF-α) levels by enzyme-linked immunosorbent assay (ELISA, R&D Systems, Minneapolis, MN, USA).

### Assessment of anti-inflammatory effect in MeT-5A cells

The mesothelial cell line MeT-5A (ATCC CRL-9444) was cultured in M199 medium (Invitrogen, Paisley, UK) supplemented with 10 % FBS, 400 nM hydrocortisone (MP Biomedicals, Solon, OH, USA), 20 mM HEPES, 870 nM insulin (Sigma-Aldrich) and 3.3 nM epidermal growth factor (ImmunoKontact, AMS Biotechnology, Oxon, UK). The cells were washed with Dulbecco’s PBS (Sigma-Aldrich) and harvested by incubation with trypsin (Invitrogen). The cell density was adjusted to 1.6 × 10^5^ cells/ml, and 100 μl of the cell suspension was added to the wells of a 96-well cell culture plate (Sarstedt). After 2 days of incubation, the cells were stimulated by adding 0.1 ng/ml interleukin 1 beta (IL-1β, R&D Systems) in the medium specified above with 10 % FBS exchanged for 5 % h.i. FBS. Titration experiments were previously performed to identify the IL-1β concentration mediating a close to maximum release of plasminogen activator inhibitor type 1 (PAI-1) over a period of 6 h. PXL150 in a concentration range from 40 to 160 μM was added directly after the addition of IL-1β. The supernatants were collected after 6 h of incubation, centrifuged for 6 min at 400 × *g* and frozen at −20 °C. The samples were kept frozen until analysed for PAI-1 levels by TriniLIZE PAI-1 Antigen ELISA (Trinity Biotech, Bray, Ireland).

### Assessment of cytotoxicity in THP-1 and MeT-5A cells

The cells were cultured, stimulated and treated with PXL150 as described above. Triton X-100 (ICN Biomedicals Inc., OH, USA) was used as a positive control and added at the same time as the peptide. After 6 h of incubation, TACS MTT (3-(4, 5-dimethylthiazol-2-yl)-2,5-diphenyltetrazolium bromide) assay (R&D Systems) was performed according to manufacturer’s instructions. In short, 10 μl of MTT reagent was added to each well, and the cell culture plate was incubated for 2 h at 37 °C. Subsequently, 100 μl of detergent reagent (100 μg/ml) was added and the plate was incubated in the dark, at room temperature overnight and the absorbance was measured at 570 nm with the reference wavelength of 650 nm.

### Evaluation of antibacterial effect in an in vivo excision wound model in rat

The ability of PXL150 to reduce the bacterial counts was estimated in an in vivo excision wound model in rats based on a previously published model (Saymen et al. 1972). All animal experiments were performed after prior approval from the local Ethics Committee for Animal Studies at the Administrative Court of Appeals in Gothenburg, Sweden. Briefly, female Sprague–Dawley rats (200–250 g, Charles River Laboratories, Sulzfeldt, Germany) were housed at the Laboratory of Experimental Biomedicine, Gothenburg, Sweden. The rats were kept in a 12-h light–dark cycle, had free access to water and pellets (Lab For, Lantmännen, Malmö, Sweden) and were cared for in accordance with regulations for the protection of laboratory animals. Bacterial suspension of MRSA (ATCC 33591) was prepared as described above, and the final bacterial concentration was adjusted to 2 × 10^9^ CFU/ml. The rats were anaesthetised during the whole experiment and the anaesthesia was induced by an intraperitoneal injection of a mixture of fentanyl (50 μg/ml, B. Braun Melsungen AG, Melsungen, Germany) and Domitor (1 mg/ml medetomidine hydrochloride, Orion Pharma Animal Health AB, Sollentuna, Sweden) (mixture of 272 μg/kg fentanyl and 545 μg/kg medetomidine hydrochloride). The backs of the rats were shaved and swabbed with 70 % chlorhexidine alcohol. Six 10 mm × 10 mm full thickness wounds were made on the back of each animal, separated by a distance of 5 mm. Immediately after surgery, the wounds were seeded with 20 μl of the bacterial suspension. Two hours post-surgery, 100 μl of PXL150 in H_2_O (in a concentration range of 0.1–2 mg/ml) or placebo (H_2_O) was added to each wound. The treatment was randomised for all wounds. The animals were euthanised 2 h post-treatment by an overdose of pentobarbital sodium (Pentobarbital vet, APL, Stockholm, Sweden). The whole wound area was dissected, transferred to a microcentrifuge tube and placed on ice. Five hundred microliters of Kligman buffer (0.1 % Triton X-100 in 0.075 M phosphate, pH 7.9) was added and the tube was vortexed for 2 min, followed by shaking for 10 min at 1,400 rpm. Each suspension was diluted in four tenfold serial steps in diluted Kligman buffer (0.05 % Triton X-100 in 0.0375 M phosphate). Fifty microliters from each dilution was seeded on blood agar plates and incubated at 37 °C overnight. Plates containing 30–300 CFU were counted. All personnel involved in the surgery, harvesting the bacteria and counting the plates, were blinded to the different treatments.

In the assessment of kinetics of the antibacterial effect of PXL150, the wounds were made and infected as described above. Two hours post-surgery, 50 μl of PXL150 in H_2_O (0.5 or 5 mg/ml) or placebo (H_2_O) was added to each wound, and samples were collected at six different time points (before treatment and 0.5, 1, 2, 3 and 4 h post-treatment) from each wound. At sampling, 25 μl of 0.037 % BHI was added to each wound, followed by gentle scraping with a plastic loop and then transferring 5 μl from the wound to 50 μl 0.037 % BHI. Sample analysis was performed as described above.

### Evaluation of antibacterial effect in an ex vivo wound model using pig skin

The ability of PXL150 to reduce colonisation of *S. aureus* (ATCC 12600) was evaluated in an ex vivo wound model using pig skin as previously described (Schmidtchen et al. 2009). The pigs used in the study were a breed mixed of Yorkshire, Hampshire and Swedish pigham. After euthanisation, the pig was shaved and the skin was taken from the back of the pig, packed in plastic foil and frozen at −20 °C for storage. When the experiment was performed, the skin was thawed and the subcutaneous fat removed with a scalpel. The skin was placed in a petri dish with Kleenex paper tissue in the bottom, soaked in sterile H_2_O. The pig skin was cleaned with 70 % ethanol and punch biopsies were made, approximately 0.5–1 mm in thickness and 3 mm in diameter. The top cylinder of a cut 1.5 ml microcentrifuge tube (diameter approximately 9 mm) was glued around each punch wound with ethyl cyanoacrylate glue (Loctite super glue gel, Henkel Norden, Stockholm, Sweden). The area inside the cylinder was washed twice with 250 μl sterile H_2_O.

Bacteria were cultured as described above and diluted to a concentration of 10^7^ CFU/ml. One hundred microliters of the bacterial suspension was applied to each wound area. The lid of the petri dish was applied to create a moist chamber, and the dish was incubated for 2 h at 37 °C. One hundred microliters of the peptide solution diluted in H_2_O (in a concentration range of 0.1 to 2 mg/ml) or placebo (H_2_O) was added to the infected areas and the petri dish was incubated for another 4 h at 37 °C. The liquid in the cylinder was discarded and the bacteria were harvested by adding 210 μl of Kligman buffer (0.1 % Triton X-100 in 0.075 M phosphate, pH 7.9), followed by scratching the area inside the cylinder with a plastic loop. The suspension was transferred to a new tube, the procedure was repeated once and the two fractions of liquid from the infected areas were pooled. The suspensions were diluted in five tenfold steps in diluted Kligman buffer (0.05 % Triton X-100 in 0.0375 M phosphate) and then seeded on blood agar plates and incubated at 37 °C overnight. The plates containing 30–300 CFU were counted. All personnel involved in the surgery, harvesting the bacteria and counting the plates, were blinded to the different treatments.

### Preliminary assessment of systemic toxicity

PXL150 at the concentrations of 1.25 mg/ml (2.5 mg/kg) and 6.25 mg/ml (12.5 mg/kg) or vehicle (0.9 % sodium chloride) was administrated as a single subcutaneous injection at the neck region of the rats (Sprague–Dawley, 200–300 g) in a volume of 0.2 ml per 100 g body weight. The study involved six rats divided into three groups as listed above with two rats per group. Observations of the rats’ general health condition were performed 0.5, 2, 4, 24 and 48 h post-treatment by monitoring general behaviour and clinical signs such as body posture (recumbent position or lying on side), central excitation (twitches, tremor or seizures), mood/awareness (vocalisation or restlessness), motor activity (absence of spontaneous locomotor activity) and autonomic profile/miscellaneous (abdominal contortion, increased salivation, piloerection or diarrhea).

### Statistical analysis

The data are presented as the percentage of the control group ± SEM. The results were analysed with unpaired Student’s *t* test, with a value of *p* < 0.05 considered statistically significant.

## Results

### In vitro microbicidal effect of PXL150

The concentration of PXL150 needed for killing ≥99 % of bacteria, MMC_99_, was examined against a representative set of microorganisms, appearing in topical and parenteral infections including Gram-positive bacteria (*S. aureus*, MRSA, *S. pyogenes*, *S. epidermidis*, and *P. acnes*), Gram-negative bacteria (*E. coli*, *P. aeruginosa*, multiresistant isolates of *K. pneumoniae*, and *A. baumannii*) and the yeast species *C. albicans*, *C. parapsilosis*, *C. glabrata* and *C. krusei.* The conventional antibiotics used for topical treatment of infections, mupirocin and fusidic acid, were included as control substances. PXL150 demonstrated a broad spectrum of microbicidal activity against the Gram-positive and Gram-negative bacteria and the yeast with similar concentration of peptide needed for ≥99 % killing of microorganisms (MMC_99_ = 3–6 μg/ml, Table 1), except for *C. glabrata* where 12.5 μg/ml of PXL150 was needed. The microbicidal effect of PXL150 on MRSA and on a fusidic acid-resistant strain from a freshly collected clinical isolate of *S. aureus* was identical to the corresponding control strain tested (MMC_99_ = 3 μg/ml). Mupirocin and fusidic acid demonstrated an effect only against Gram-positive bacteria, with ≥99 % killing of microorganisms at the same concentration range as for PXL150, except for fucidic acid, which failed to kill *S. pyogenes.* The antibiotic controls showed no effect against Gram-negative bacteria or yeast (Table 1). The MMC assay was performed in diluted BHI, which is the most commonly used media in this type of assessment. To evaluate the microbicidal effect of PXL150 in a wound-like environment, the MMC_99_ assay against two main pathogens in SSTIs—*S. aureus* and MRSA—was complemented with experiments in 50 % h.i. SWF. The MMC_99_ values were increased under these conditions; however, a concentration of 50 μg/ml of PXL150 was sufficient for killing ≥99 % of both strains. The antibiotic controls were not sensitive to the high salt media (Table 1).

**Table 1:** Antimicrobial effect of PXL150 was evaluated in MMC_99_ assay against a panel of Gram-positive and Gram-negative bacterial strains and yeast strains in 0.037 % BHI or 50 % h.i. SWF

Strain of microorganism	PXL150	Fusidic acid	Mupirocin
0.037 % BHI, MMC_99_ (μg/ml)			
*S. aureus* (ATCC 12600)	3.1	6.3	3.1
MRSA (ATCC 33591)	3.1	6.3	3.1
*S. pyogenes* (ATCC 12344)	3.1	>200	6.3
*S. epidermidis* (ATCC 12228)	3.1	6.3	3.1
*P. acnes* (ATCC 6919)	6.3	nd	>200
*E. coli* (ATCC 11775)	6.3	>200	>200
*P. aeruginosa* (ATCC 15442)	6.3	>200	>200
*K. pneumoniae* (ATCC 13883)	6.3	>200	>200
*A. baumannii* (ATCC 19606)	3.1	>200	>200
*C. albicans* (ATCC 64549)	3.1	>200	>200
*C. parapsilosis* (ATCC 22019)	6.3	nd	nd
*C. krusei* (ATCC 6258)	6.3	nd	nd
*C. glabrata* (CCUG 35267)	12.5	nd	nd
50 % h.i. SWF, MMC_99_ (μg/ml)			
*S. aureus* (ATCC 12600)	50	6.3	3.1
MRSA (ATCC 33591)	50	6.3	3.1

The substance was tested in concentrations up to 200 μg/ml. PXL150 was added to the bacterial cells in duplicate (*n* = 2). Data are presented as maximum values from at least two independent experiments

*h.i.* heat-inactivated, *nd* not determined

### Assessment of hemolytic activity of PXL150

Measurement of hemolytic activity against human red blood cells is the most common method to study the selectivity of AMPs for bacteria versus mammalian cells. In our hands, PXL150 did not display any significant hemolytic activity at concentrations up to 200 μg/ml (2–4 % hemolysis at 200 μg/ml of PXL150 observed in two independent experiments).

### Resistance development against PXL150

The potential for resistance development against PXL150 was evaluated for *S. aureus* and MRSA using a multistep dilution assay and compared to the reference antibiotic gentamicin. For both *S. aureus* and MRSA, the concentration of gentamicin needed to kill ≥99 % of the bacteria increased 16-fold during the incubation time: for *S. aureus*, the concentration increased from 0.047 to 0.75 μg/ml during the 12 days of incubation, and for MRSA, the increase was from 0.375 to 6 μg/ml during 21 days of incubation. However, the MMC_99_ values for PXL150 over the 21 days of incubation with *S. aureus* remained stable at 3 μg/ml, while the values for MRSA varied between 1.5 and 3 μg/ml, which are within the limits of assay variation.

### Cytoplasmic membrane depolarisation of target bacteria by PXL150

**Fig. 1:**
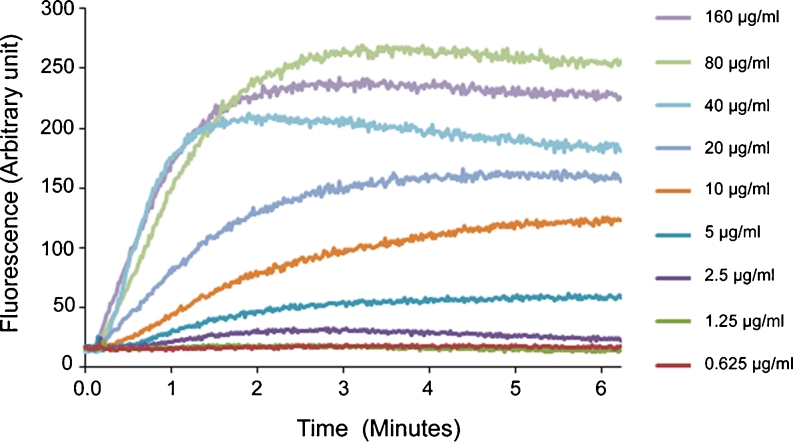
Effect of PXL150 on the fluorescence intensity as a measurement of depolarisation of the bacterial cell membrane. PXL150 in a concentration range of 0.625 to 160 μg/ml was added to a suspension of *S. aureus* supplemented with 100 mM KCl and 0.4 mM DiSC3(5). The fluorescence emitted was registered continuously at 670 nm until the fluorescence was stabilised

To characterise the mode of action of killing target bacteria, PXL150-induced changes in the cytoplasmic membrane of *S. aureus* were assessed using the membrane potential-sensitive cyanine dye DiSC_3_(5). Addition of PXL150 to the bacteria resulted in a rapid increase in fluorescence, consistent with the release of dye due to the dissipating membrane potential by PXL150. The fluorescence increase profile indicates that membrane depolarisation is dependent on peptide concentration as well as time (Fig. 1). The fluorescence intensity increased in a dose–response relationship in the concentration range of 2.5 to 80 μg/ml of PXL150 where the signal corresponding to 80 μg/ml equals the fluorescence signal of the dye in buffer only (Online Resource 1). The maximum depolarisation for all peptide concentrations used occurred within approximately 2 to 5 min after the addition of PXL150. The rate at which the fluorescence intensity increased over time, i.e. the slope of the curve, augmented from 2.5 up to 40 μg/ml of PXL150, where it was stabilised (Fig. 1).

**Fig. 2:**
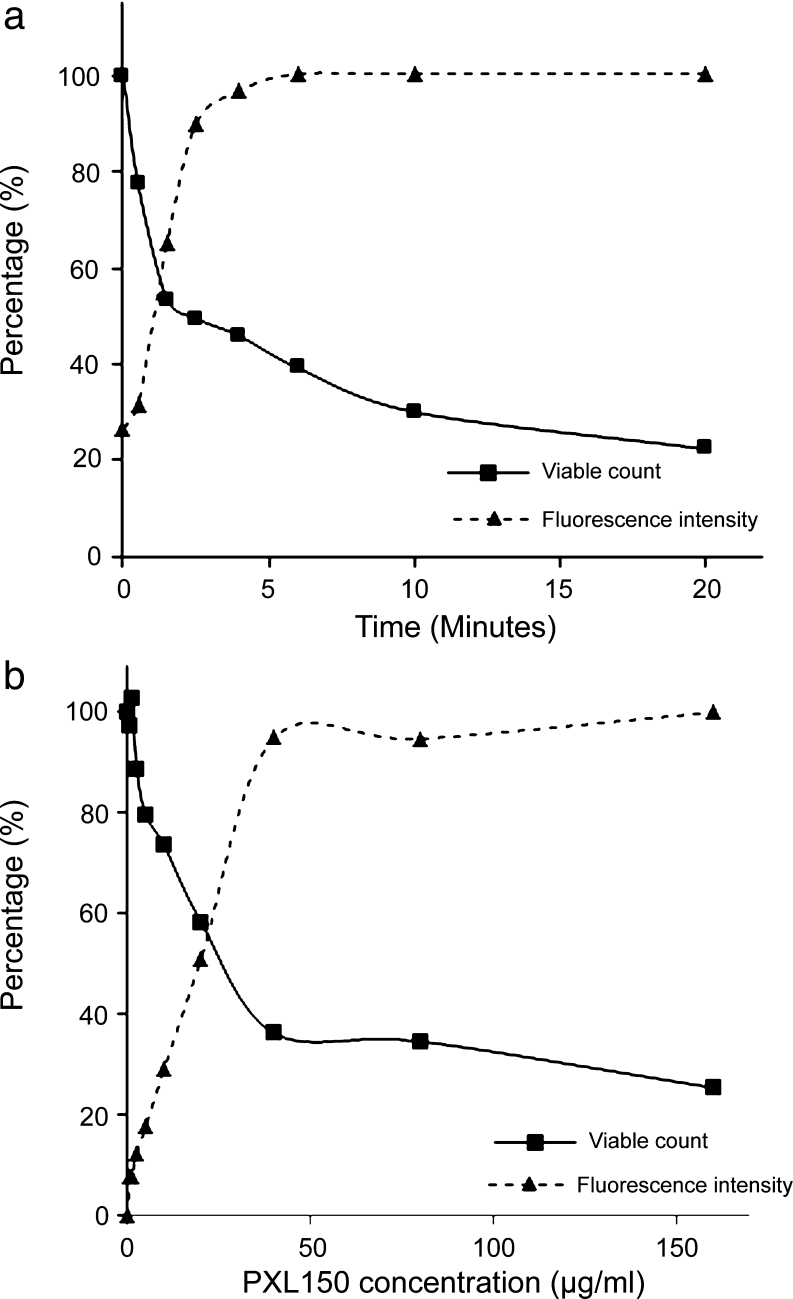
Correlation between membrane depolarisation and killing of target bacteria by PXL150. **a** The fluorescence intensity and bacterial viable counts were monitored at indicated time points after the addition of 5 μg/ml PXL150 to a bacterial suspension supplemented with 100 mM KCl and 0.4 mM DiSC3(5). The maximum polarisation intensity of 5 μg/ml PXL150 or bacterial counts at time 0 was set to 100 %. **b** The fluorescence intensity and bacterial viable counts were monitored at 1.5 min post-addition of PXL150 in a concentration range of 0.625 to 160 μg/ml to a bacterial suspension supplemented with 100 mM KCl and 0.4 mM DiSC3(5). The maximum fluorescence intensity of 160 μg/ml PXL150 at the 1.5-min time point or bacterial counts without peptide added was set to 100 %

To describe the correlation between membrane depolarisation effect and microbicidal properties of PXL150, the bacterial viability was measured in samples taken at different time points after the addition of the peptide directly from the assay tube with the concentration of 5 μg/ml of PXL150. This concentration of PXL150 did not result in complete elimination of the bacteria at any of the time points evaluated. However, there was an obvious correlation between the time required for cytoplasmic membrane depolarisation and kinetics of killing the bacteria, where the 5-min time point corresponded to close to maximal depolarisation as well as to approximately 80 % of maximal microbicidal effect (Fig. 2a). Moreover, the viability was assessed at all concentrations of PXL150 1.5 min after the addition of the peptide (Fig. 2b). Again, a close correlation between concentrations causing membrane depolarisation and microbicidal effect was seen with peptide concentrations of 40 μg/ml resulting in 95 % of maximal fluorescence reading while leading to approximately 90 % of the maximal reduction in bacterial counts.

### Anti-inflammatory activity of PXL150 in THP-1 and MeT-5A cell lines

**Fig. 3:**
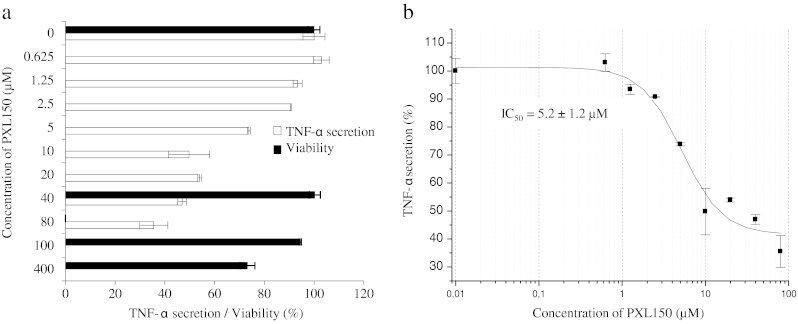
Effect of PXL150 on TNF-α secretion and viability of LPS-induced PMA-treated THP-1 cells. The peptide was added to cells in triplicate (*n* = 3) 30 min after the addition of LPS (0.1 ng/ml). Cytokine levels were measured in the cell supernatants by ELISA after 6 h of stimulation. **a** TNF-α secretion (*white bars*) is presented as relative secretion (%) ± SEM, with stimulated cytokine levels without peptide added set to 100 %. Viability of THP-1 cells (*black bars*) is presented as relative viability (%) ± SEM, with viability in stimulated cells without peptide set to 100 %. The cell viability was only measured for the peptide concentrations of 0, 40, 100 and 400 μM. **b** Data were fitted to the dose–response curve using a four-parameter fit in the Origin software. The IC_50_ value was automatically calculated by the software Origin

**Fig. 4:**
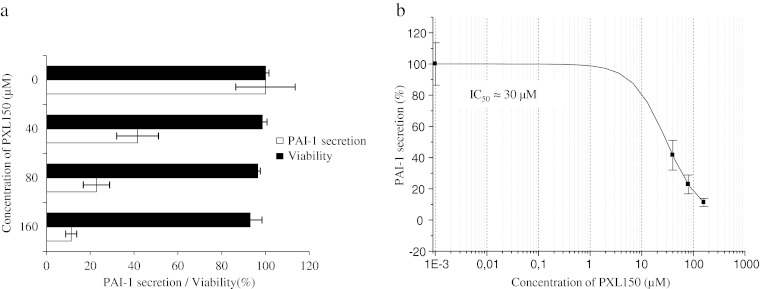
Effect of PXL150 on PAI-1 secretion and viability of IL-1β-induced MeT-5A cells. The peptide was added to cells in triplicate (*n* = 3) after the addition of IL-1β (0.1 ng/ml). Cytokine levels were measured in the cell supernatants by ELISA after 6 h of stimulation. **a** PAI-1 secretion (*white bars*) is presented as relative secretion (%) ± SEM, with stimulated cytokine levels without peptide added set to 100 %. Viability of MeT-5A cells (*black bars*) is presented as relative viability (%) ± SEM, with viability in stimulated cells without peptide set to 100 %. **b** Data were fitted to the dose–response curve using a four-parameter fit in the Origin software. The IC_50_ value was automatically calculated by the software. Due to an unsatisfactory curve fitting, IC_50_ was estimated to be approximately 30 μM

The anti-inflammatory effect of PXL150 was studied in macrophages derived from the human monocyte cell line THP-1 and in the human mesothelial cell line MeT-5A after stimulation with LPS and IL-1β, respectively. The secretion of the most commonly used inflammation marker TNF-α and acute phase protein during the first phases of inflammation, PAI-1, was analysed using ELISA. PXL150 showed pronounced anti-inflammatory effect in a low micromolar range by inhibiting secretion of both TNF-α (Fig. 3) and PAI-1 (Fig. 4) with a half maximal inhibitory concentration (IC_50_) of 5.2 ± 1.2 μM and approximately 30 μM, respectively. In the experiment using THP-1 cells, the peptide was added to the cell culture medium 30 min after the addition of LPS. Based on previous findings, this time interval should be sufficient for LPS to bind to the cell receptor and, thus, to exclude that the peptide would neutralise the effect of LPS on cytokine production only by scavenging this agent (Elass-Rochard et al. 1998; Haversen et al. 2000).

Next, we examined the cytotoxicity against both cell types, assessing the effects on cell growth by measuring the mitochondrial reduction of MTT to a coloured product by live cells. In both cell types, the viability was largely unaffected (higher than 90 % of total number of cells) at concentrations of 100 μM PXL150 or higher (Figs. 3 and 4), indicating that cytotoxicity had not significantly contributed to the reduction in cytokine secretion observed.

### Antibacterial effect of PXL150 in animal models of topical infection

**Fig. 5:**
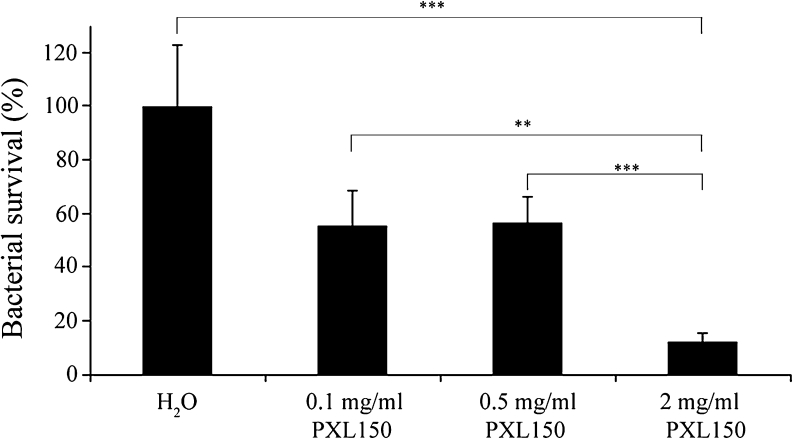
In vivo bacterial clearance of MRSA from experimental excision wounds in rats after treatment with PXL150. Wounds infected with MRSA were treated with the PXL150 concentrations of 0.1, 0.5 and 2 mg/ml, or placebo (H_2_O), and samples were harvested 2 h after the treatment. Results are presented as relative bacterial survival (%) compared to placebo ± SEM (*n* = 15 wounds). **p < 0.01, ***p < 0.001

**Fig. 6:**
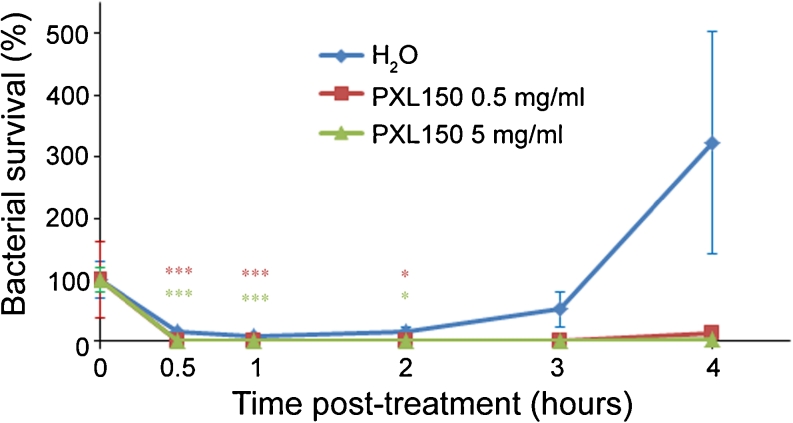
Assessment of kinetics of bacterial clearance of MRSA from experimental excision wounds in rats by PXL150. Wounds infected with MRSA were treated with the PXL150 concentrations of 0.5 and 5 mg/ml, or placebo (H_2_O). The viable counts were assessed 0.5, 1, 2, 3 and 4 h post-treatment in the same wound. Results are presented as relative bacterial survival (%) compared to placebo ± SEM (*n* = 15 wounds). *p < 0.05, ***p < 0.001, where comparison between the placebo group and 0.5 mg/ml of PXL150 is indicated by *red symbols* while the comparison between placebo group and 5 mg/ml of PXL150 is indicated by *green symbols*

**Fig. 7:**
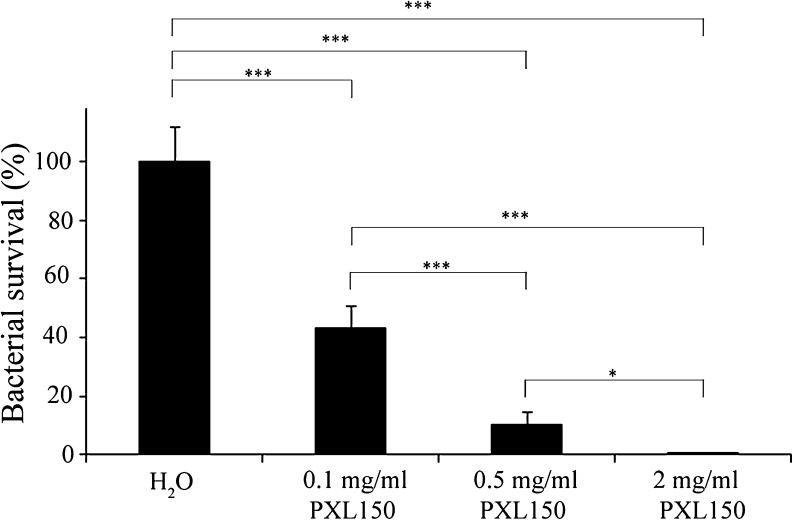
Ex vivo bacterial clearance of *S. aureus* from infected pig skin wounds after treatment with PXL150. Wounds infected with *S. aureus* were treated with PXL150, in the concentrations of 0.1, 0.5 and 2 mg/ml, or placebo (H_2_O), and samples were harvested 4 h after the treatment. Results are presented as relative bacterial survival (%) compared to placebo ± SEM (*n* = 10 wounds). *p < 0.05, ***p < 0.001

The antimicrobial effect of PXL150 was investigated by dose–response studies in two different animal models of topical infection, an in vivo excision wound model in rats and an ex vivo pig skin model. PXL150 dissolved in H_2_O at the concentrations of 0.1, 0.5 and 2 mg/ml was used in both models. In the in vivo excision wound model in rats, PXL150 was administered to MRSA-infected full thickness wounds 2 h post-infection, and the bacteria were harvested 4 h post-infection. The highest concentration of PXL150 of 2 mg/ml reduced the bacterial survival with 88 % compared with placebo (H_2_O), and there was a statistically significant difference comparing the concentration of 2 mg/ml to the placebo as well as to the two lower concentrations of 0.1 and 0.5 mg/ml of PXL150 (Fig. 5). Complementary to these experiments, the kinetics of the antibacterial effect of PXL150 was assessed in the same model with viable counts of bacteria analysed in the wound at 0.5, 1, 2, 3 and 4 h post-treatment. Administration of 0.5 and 5 mg/ml of PXL150 resulted in an instant antibacterial effect with reduction of bacterial survival of 99 and >99.9 %, respectively, compared with placebo at 0.5 h post-treatment. The effect sustained for at least 4 h after treatment as only 3 % of CFUs were recovered at this point in rats treated with 5 mg/ml of PXL150, compared with the placebo group (Fig. 6). The antibacterial effect of PXL150 was confirmed in an ex vivo pig skin model in which PXL150 was administered 2 h post-infection with *S. aureus*, and the bacteria were harvested 6 h post-infection. All concentrations tested significantly reduced the bacterial survival, and the concentration of 2 mg/ml reduced the bacterial survival with ≥99 % compared with the placebo. There were significant differences between all concentrations demonstrating a clear dose–response relationship of the effect of PXL150 (Fig. 7).

### Preliminary toxicology assessment of PXL150

Potential systemic toxicity of PXL150 was studied in rats with 2.5 and 12.5 mg/kg of PXL150, or vehicle (0.9 % sodium chloride) administered by a single subcutaneous injection in two rats per dose group. Clinical signs were monitored 0.5, 2, 4, 24 and 48 h post-treatment. During the period of observation, there were no behavioural signs seen in any of the groups suggesting no systemic toxicity. Moreover, no treatment-related local adverse effects were observed in the infected in vivo excision wound model in rats described above.

## Discussion

AMPs are well recognised for their pivotal role in preventing infections by microbial pathogens in many organisms (reviewed in Hancock and Sahl 2006). Today, at least 15 AMPs or peptide mimetics are being tested in clinical trials as antimicrobial or immunomodulatory agents (Fjell et al. 2012), to prove their usefulness as a new class of anti-infectious drugs.

Here we describe a novel AMP, PXL150, which demonstrates in vitro a broad spectrum microbicidal action with activities in the micromolar range against both Gram-positive and Gram-negative bacteria. PXL150 showed pronounced effect against MRSA at concentrations identical to the ones killing the *S. aureus* control strain. Based on the models of mechanism of action of AMPs, they are believed to initially interact with microbial membranes heavily populated by negatively charged phospholipids by electrostatic forces. Thus, physiological conditions with high salt concentration are expected to weaken the electrostatic charge interactions and lower the efficacy of the AMP (Marr et al. 2006). The activity of PXL150 was indeed reduced in the incubation mixture of serum containing SWF; however, even under these conditions, a potent action against both *S. aureus* and MRSA was observed. Importantly, the potent microbicidal activity and broad antibacterial spectrum of PXL150 were not associated with any hemolytic activity, indicating dissociation between antibacterial and antieukaryotic activities, i.e. a high therapeutic index.

Resistance development is considered the most important factor affecting the antibacterial market, in both the community and hospital settings, and it is a particular problem for the treatment of SSTIs (Datamonitor 2009). Interestingly, bacteria have been exposed to AMPs for millions of years, and despite the continual presence of AMPs in bacterial environment, widespread resistance has not been reported. Therefore, development of complete resistance against AMPs has been proposed to be unlikely (Marr et al. 2006). This view is further supported by the mode of action of AMPs, which unlike antibiotics, involves attacking multiple targets, and thus, the elimination of any one target by the host is of lesser consequence (Fjell et al. 2012). We evaluated if resistance to PXL150 would evolve under continued selection. During 21 passages of *S. aureus* and MRSA in sub-MIC concentration of the peptide, no indication of resistance development was observed, whereas under the same conditions, resistance to the conventional antibiotics gentamicin increased by 16-fold. These data indicate that under the experimental conditions adopted, direct selection did not lead to resistance development against PXL150.

Cytoplasmic membrane permeabilisation leading to the dissolution of the proton motive force and leakage of essential molecules has been implicated as one mode of action for AMPs in killing both Gram-positive and Gram-negative bacteria. Ample evidence shows that membrane disruption occurs by AMPs in model membrane systems; however, the measurement of the disruption of the cytoplasmic membrane by AMPs in live bacteria has only started to emerge (reviewed by Hancock and Rozek 2002; Toke 2005). In this study, the effect of PXL150 on the cytoplasmic membrane of intact *S. aureus* was investigated using membrane potential-sensitive dye DiSC_3_(5). The fluorescence values rapidly increased when PXL150 was added to the bacteria, indicating quick membrane depolarisation by this peptide. The increase in fluorescence was concentration dependent up to 80 μg/ml of PXL150, and the maximum depolarisation occurred within 2 to 5 min after the treatment in a concentration-dependent manner. The time reported for different AMPs to reach a maximal fluorescence and, thus, to fully depolarise the cytoplasmic membrane of target bacteria is highly variable ranging from a few minutes up to 60 min (Nakajima et al. 2003; Wu and Hancock 1999), thus placing PXL150 into the group of AMPs causing a very rapid change in membrane potential. Depolarisation of the cytoplasmic membrane per se is not necessarily lethal for bacteria, e.g. ionophore valinomycin at the concentrations causing membrane depolarisation is only bacteriostatic and not bactericidal (Friedrich et al. 2000). Interestingly, several AMPs are known to have intracellular targets (Friedrich et al. 2001; Kragol et al. 2001; Xiong et al. 1999), and therefore, membrane depolarisation may be an intermediate step in the uptake of peptide rather than primary target for bacterial killing, which would implicate a lag time between depolarisation and killing. In case of PXL150, there was an obvious correlation between the increase in fluorescence intensity and bacterial killing over time as well as over the concentration range of peptide tested, indicating that membrane depolarisation is one mechanism for PXL150 to kill target bacteria. It might seem controversial that the concentration of PXL150 causing 99 % killing of *S. aureus* in MMC_99_ assay (Table 1) resulted in only minor reduction in viability when assessed in connection to membrane depolarisation assay. Moreover, even the concentration of PXL150 as high as 160 μg/ml did not result in complete elimination of the bacteria when monitored at 1.5 min post-addition of the peptide (Fig. 2b). The observation of reduced microbicidal effect of AMPs assessed in membrane depolarisation assay has been reported by several authors and relates to the reduction in the antimicrobial efficacy of AMPs in the presence of non-physiological high KCl concentration of 100 mM, as was used in membrane depolarisation assay to equilibrate the cytoplasmic and external potassium ion concentrations (Friedrich et al. 2000; Wu and Hancock 1999).

Recently, several AMPs have been reported to possess immunomodulatory functions, except their antimicrobial action (reviewed in Yeung et al. 2011). Interestingly, our study indicated that PXL150 has anti-inflammatory effect in two human cell lines. Anti-inflammatory properties might be of additional benefit in the treatment of conditions where excessive inflammatory reaction is part of the pathophysiology of the disease, such as secondarily infected dermatitis.

The therapeutic potential of PXL150 in the treatment of topical infections was evaluated in an in vivo model of full thickness wounds infected with MRSA in rats and an ex vivo model of pig skin infected with *S. aureus*. PXL150 demonstrated marked and statistically significant effect on reducing bacterial counts in both models with a dose–response relationship. A kinetic study performed in the rat model indicated that the antibacterial effect of PXL150 was very rapid with >99.9 % reduction of bacterial survival in the wounds treated with 5 mg/ml of PXL150 30 min after the peptide administration, and the effect sustained for at least 4 h post-treatment.

Mechanistically, the action of AMPs centres on membrane interaction, and therefore, the systemic toxicity is generally considered a likely risk when using these peptides, although not many studies have been published describing the toxicological findings after administration of AMPs. To evaluate the systemic safety profile of PXL150, high doses of the peptide (2.5 and 12.5 mg/kg) were administrated subcutaneously to rats, and the systemic effects were monitored at different time points post-treatment for up to 48 h. The doses of PXL150 used were 50 and 250 times higher than the expected clinical dose for the treatment of SSTIs in adults (0.05 mg/kg in a patient of 60 kg). These doses of the peptide did not lead to any behavioural effects at any of the time points followed. These results in combination with the fact that no signs of local adverse effects were observed in infected excision wound model in rats indicate that PXL150 is safe and well tolerated. It has to be notified that the number of animals included in this preliminary safety assessment was very limited and the toxicology studies including higher number of rodent as well as non-rodent species are warranted to fully characterise the safety profile of this peptide.

Dramatic increase in bacterial resistance against conventional antibiotics complicates treatment options and decisions for physicians, making the empirical treatment of SSTIs more difficult, and even dangerous, in case the drug has a narrow spectrum and is not potent against resistant strains. PXL150 may constitute a new therapeutic alternative for topical treatment potentially providing significant advantages compared to conventional antibiotics due to broad spectrum activity against both Gram-positive and Gram-negative bacteria including MRSA, combined with low potential for resistance development. The potent anti-infective effect of PXL150 was proven in two different animal models of topical wounds, while no signs of systemic toxicity were observed after subcutaneous administration of the peptide. This investigation warrants further clinical assessment to explore the potential of PXL150 as novel anti-infective agent in the treatment of SSTIs.

## Electronic supplementary material

Below is the link to the electronic supplementary material.

ESM 1 (PDF 1130 kb)
