# Supplementary material for: The novel antimicrobial peptide PXL150 in the local treatment of skin and soft tissue infections
Source: Appl Microbiol Biotechnol. 2012 Oct 4;97(7):3085–96. doi: 10.1007/s00253-012-4439-8 (PMC3602619; doi:10.1007/s00253-012-4439-8)
Supplement: Supplementary file 1 — (PDF 1130 kb) [file 253_2012_4439_MOESM1_ESM.pdf]

The novel antimicrobial peptide PXL150 in the local treatment of skin and soft tissue infections

Applied Microbiology and Biotechnology

Emma Myhrman[1], Joakim Håkansson[1], Kerstin Lindgren[1], Camilla Björn[1], Veronika Sjöstrand[1] and Margit Mahlapuu[1]

[1]Pergamum AB, Arvid Wallgrens Backe 20, 413 46 Gothenburg, Sweden

margit.mahlapuu@pergamum.com

a

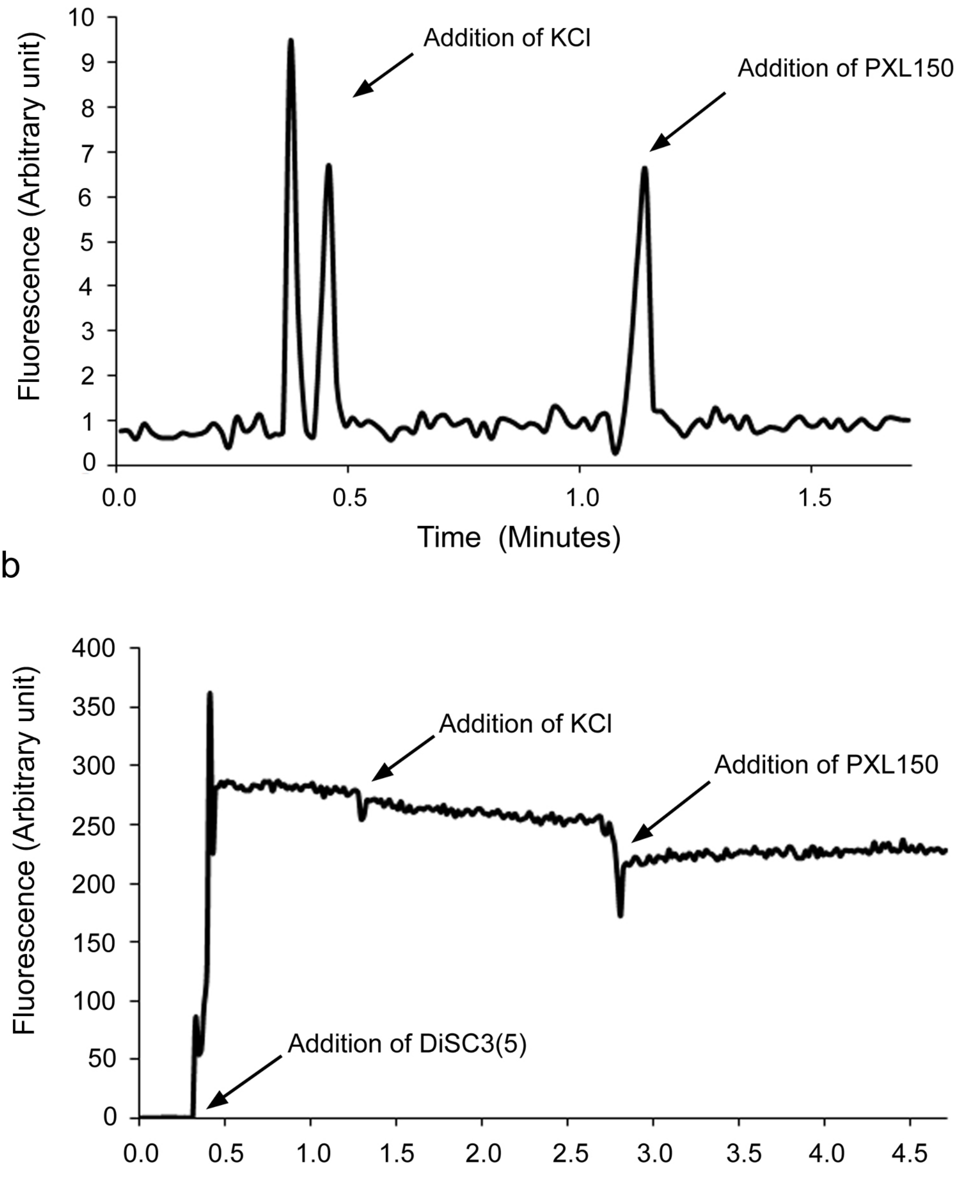

Online resource 1
(a) Assessment of auto-fluorescence of Tris buffer, supplemented with 100 mM KCl and supplemented with PXL150 160 µg/ml at 670 nm.(b) Assessment of inhibition of the fluorescence of DiSC3(5) in Tris buffer after addition of 100 mM KCl and addition of PXL150 160 µg/ml at 670 nm.
